# Systematic data-querying of large pediatric biorepository identifies novel Ehlers-Danlos Syndrome variant

**DOI:** 10.1186/s12891-016-0936-8

**Published:** 2016-02-16

**Authors:** Akshatha Desai, John J. Connolly, Michael March, Cuiping Hou, Rosetta Chiavacci, Cecilia Kim, Gholson Lyon, Dexter Hadley, Hakon Hakonarson

**Affiliations:** Center for Applied Genomics, The Children’s Hospital of Philadelphia, Philadelphia, 3615 Civic center Blvd, Philadelphia, PA 19104 USA; Department of Pediatrics at the Perelman School of Medicine, University of Pennsylvania, Philadelphia, PA 19104 USA

**Keywords:** Whole genome sequencing, Ehlers-Danlos Syndrome, Biorepository, Electronic medical records, EDS, EMR, WGS

## Abstract

**Background:**

Ehlers Danlos Syndrome is a rare form of inherited connective tissue disorder, which primarily affects skin, joints, muscle, and blood cells. The current study aimed at finding the mutation that causing EDS type VII C also known as “Dermatosparaxis” in this family.

**Methods:**

Through systematic data querying of the electronic medical records (EMRs) of over 80,000 individuals, we recently identified an EDS family that indicate an autosomal dominant inheritance. The family was consented for genomic analysis of their de-identified data. After a negative screen for known mutations, we performed whole genome sequencing on the male proband, his affected father, and unaffected mother. We filtered the list of non-synonymous variants that are common between the affected individuals.

**Results:**

The analysis of non-synonymous variants lead to identifying a novel mutation in the *ADAMTSL2* (p. Gly421Ser) gene in the affected individuals. Sanger sequencing confirmed the mutation.

**Conclusion:**

Our work is significant not only because it sheds new light on the pathophysiology of EDS for the affected family and the field at large, but also because it demonstrates the utility of unbiased large-scale clinical recruitment in deciphering the genetic etiology of rare mendelian diseases. With unbiased large-scale clinical recruitment we strive to sequence as many rare mendelian diseases as possible, and this work in EDS serves as a successful proof of concept to that effect.

## Background

Ehlers Danlos Syndrome (EDS) describes a group of heritable disorders where collagen synthesis and fibril formation is disrupted. It affects approximately 1 in 5000 to 1 in 100,000 individuals, depending on subtype [1, 2]. Predominant phenotypes include articular hypermobility, lax joints, and fragile tissue, skin, and blood vessels. EDS can be extremely debilitating, requiring preventative and protective measures from birth or onset of symptoms. A large number of subtypes have been described, including six major classifications: 1) Classical (Types I&II); 2) Hypermobile (Type III); 3) Vascular (Type IV); 4) Kyphoscoliosic (Type VI); 5) Arthrochalasic (Type VIIA &VIIB); and 6) Dermatosparaxic (Type VIIC) [3]. These can be transmitted through autosomal dominant, autosomal recessive, or x-linked inheritance. Mutations in a number of genes involved in collagen synthesis have been associated with different EDS subtypes, including *COL1A1*, *COL1A2, COL5A1, COL5A2* [2, 3], and *ADAMTS2*^21^. All EDS Type VIIC mutations identified to date have been autosomal recessive. Here, we report on an EDS patient, with symptomology that includes dermatosparaxis (literally, “tearing of the skin”), which is characteristic of EDS Type VIIC.

## Methods

### Recruitment

The proband (and sister) were recruited at The Center for Applied Genomics at The Children’s Hospital of Philadelphia under a large-scale clinical recruitment project, *A Study of the Genetic Causes of Complex Pediatric Disorders, aimed at recruiting over 100,000 Children with both common and rare medical disorders*. This study was approved by the institutional review board of The Children’s Hospital of Philadelphia. The proband and his family members consented to release of de-identified medical and genomic information for publication. For the proband and sister, this was in the form of parental consent. All study participants blood was drawn when they presented for a clinic visit. They consented to prospective genomic analysis and analysis of electronic medical records (EMRs). Recruitment was not targeted, and the patients came to our attention through systematic data querying of de-identified EMR records from over 80,000 individuals recruited over the last six years. Cross-referencing our biorepository with rare-disease codes (as defined by Online Mendelian Inheritance in Man (OMIM) code 225410 and International Statistical Classification of Diseases and Related Health Problems 9 (ICD9) code 756.83), we identified the EDS proband who had longitudinal EMRs that included a detailed family history and relatively extensive medical history of both parents.

### EMR querying

Our broad consent protocol includes extraction of DNA, blood/sera, access to EMRs, and the permission to perform a wide range of genomic studies in consenting individuals. The EMR product, which captures over one million clinical office visits annually, is EpicCare®, (Epic Systems, WI), and includes the reason for visit, diagnosis codes (ICD-9-CM format), growth measurements, medications prescribed, and referrals made. The EMR is directly interfaced with three laboratory information systems, including one internal and two external (Quest Diagnostics, LabCorp) lab systems. All laboratory test results are available in a structured format, mapped to Current Procedural Terminology (CPT) codes, and Logical Observation Identifier Names and Codes (LOINC) as needed. All of this information is captured and moved into our repository in a structured format for consented research participants. We also receive radiology reports, surgical notes, hospital discharge summaries, and Emergency Department summaries and dictated specialist reports.

Patient data is encrypted and integrated into a custom phenotype browser, which includes all EMR, as well as additional survey data collected at recruitment (family history/diagnoses etc.). This allows a multidirectional flow of de-identified phenotype information to be directly coupled with an automated analysis pipeline, utilizing high-efficiency software tools. Only encrypted/de-identified data reaches the phenotype browser, where it can be coupled with genotyping, sequencing and other data that are available on the subjects. Of the patient DNA samples that have consented to genetic studies, we have >99 % availability of samples available for re-genotyping under CLIA/CAP certification or NGS, with the ~1 % loss mainly due to small amounts of blood being obtained from the youngest children or ineffective DNA extraction from saliva, but saliva samples constitute less than 5 % of our DNA sample sets, 95 % of which are blood-derived.

Diagnostic codes (e.g. 756.83) and keyword (e.g. “Ehlers Danlos, EDS”) keyword searches identified a list of 119 patients, all of which included access to lab and diagnostic reports as described above. Patient data was then re-identified through a reverse-encryption process, which allowed our clinical team (who have no access to genomic data) to conduct a complete review of the proband’s entire medical chart (including medical letters) and family medical history. Of these 119 patients, only in the case of the identified proband did we have access to the samples of a rather complete pedigree. For this family we had the proband being diagnosed with EDS and father and sister having EDS like phenotype the mother and maternal half-brother were normal.

### Proband’s medical history

The proband is currently nine years for age. He was delivered at full term through vaginal delivery. The birth was traumatic and forceps and suctioning were used. His birth weight was 4.1 kg. He was diagnosed with EDS at the age of 9 months, following admission for joint laxity and ankle crepitus. He had slightly delayed development, with ambulation at approximately 14 months. At two years of age, he was referred to a neurologist with amblyopia, who noted moderate gross motor delay, increased shoulder girdle weakness and central hypotonia. At 2.5 years of age, delays in his fine motor skills and speech articulation were recorded. He had difficulty chewing and had weak mouth muscles. Difficulties in fine motor skills were recorded again at five years of age, and abnormal gait, poor coordination, and developmental delay were noted at multiple time points. At 9 years-of-age, he was at the 99^th^ percentile for both height (148.4 cm) and weight (48.3 kg) based on the Center for Disease Control and Prevention (CDC) weight-for-age data. He is normocephalic, and has a fibrous anterior fontanel. He has a slightly triangular face with slight mid-face flattening. His adipose tissue in his lower jaw and neck is decreased, and he has blue sclerae. He has a normal palate. He has a I/VI heart murmur with no clicks and regular rate and rhythm.

The proband presented with a history of recurrent joint pain and was found to have increased mobility in his metacarpal joints, proximal and distal interphalangeal joints, and significant thumb hypermobility. He has increased flexibility of his wrists, while his elbows can be slightly hyperextended. He also has a hyperextensible ear. He has decreased movement in his ankles, which are also slightly contracted and unable to be dorsiflexed beyond 90^0^. Hypermobility improved from ages 3 to 9 years (current age), during which time he received extensive occupational and physical therapy. He does not have any scoliosis or kyphosis. He has had two patella dislocations, and has been admitted for fractures (distal radius and ulna buckle) and sprain (ankle). Bone density, however, is normal (z-score = 0.3 SD) as per dual-energy X-ray absorptiometry (DXA) scanning at 9 years.

Other problems include urinary incontinence, ureterectasis (hydroureter), and profound hydronephrosis. At age six years, he had two posterior urethral valve surgeries, requiring a suprapubic tube. He has a history of antibody deficiency and has been receiving intravenous immunoglobulin (IVIG) therapy every 3 weeks. He has exotropia (his left eye drifts out) and amblyopia (commonly known as “lazy eye”). He is developmentally delayed, and a diagnosis of ADHD was recorded at 7 years of age. EEG at 2 years of age was normal. “Components of autism” were recorded once, at age 7.5 years. “Sensory integration issues” were recorded once at 6 years of age. He has decreased adipose tissue, decreased muscle mass and hypotonia. He has a history of asthma with exacerbation, and reports daily fatigue. No cardiovascular abnormalities have been recorded. His medical history was otherwise significant for dysphagia, recurrent vomiting and chronic constipation.

Hitherto, no genetic anomalies have been identified. At 2.9 years, he was screened for collagen/procollagen abnormalities. None were detected – cells synthesized and secreted types I and III procollagens normally, and the efficiency of conversion of procollagen to collagen, was similar to control cells. These findings make it unlikely that he has a form of osteogenesisimperfecta (OI) [4–8], as cells from more than 85 % of individuals with clinically recognizable OI have alterations in either the amount or the structure of type I procollagen synthesized and typically result in a decrease type I procollagen production by ~50 %. OI types II (OMIM 166210), III (OMIM 259420), and IV (OMIM 166220) are also unlikely, as they are typically caused by mutations affecting the structure of the pro-alpha 1(I) and pro-alpha 2(I) chains of type I procollagen, and result in substitution for glycine residues in the triple-helical domains of these chains. These findings exclude most forms of EDS type IV, which typically results from mutations that affect the synthesis, structure or secretion of type III procollagen. They also exclude some forms of EDS type VII that are caused by genomics deletions that affect the conversion of procollagen to collagen. Genetic screening tests for mutations in known EDS genes, including *TNXB* and *COL3A1* were also negative*.*

### Family history

**Fig. 1:**
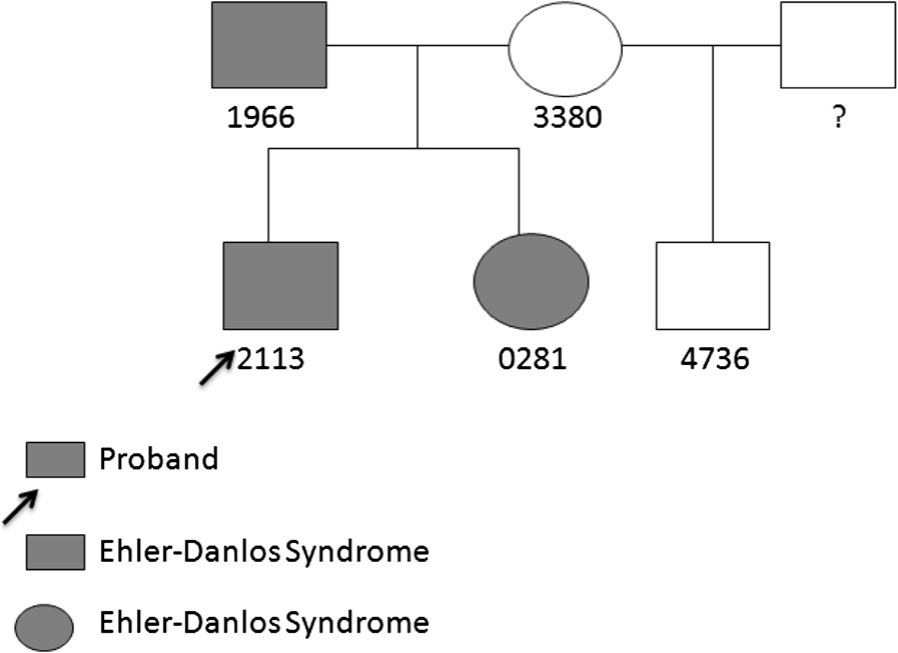
Pedigree Structure of the Family with their De-Identified IDs. Individuals with confirmed or suspected EDS are shaded. The mother (3380), father (1966) and affected male proband (2113) had their whole genome sequenced. For validation, affected sister (0281) and half-brother (4736) of the proband were Sanger sequenced with the other three family members

A complete pedigree (Fig. 1) is not available, but the proband’s family history does record several details pertinent to this study. The proband’s sister is seven years of age, and was last seen two years ago. There is evidence of EDS with incomplete expression, with relevant (endo) phenotypes including hypermobility, slow-healing scars, and urinary incontinence. Ultrasound of kidney and bladder reveal no structural abnormalities. Likewise, cardiovascular functions appear normal, and osseous structures are unremarkable.

We do not have direct access to the medical records of the father, but a detailed family history of the proband reveals evidence of an EDS-type phenotype. The father has had multiple joint dislocations, wounds that did not heal well, and multiple minimum trauma fractures. He had shoulder surgery following multiple dislocations.

The proband has a paternal uncle with suspected EDS, including skin elasticity, and poor healing. He also has a history of multiple shoulder dislocations and fractures with trauma. A paternal aunt is also mentioned in the family history, which evidences hypermobility (reportedly able to make a “praying sign behind her back”). She also has a history of resting tremors, and poor skin healing. The proband’s paternal grandmother has several EDS-like symptoms, including blue sclera, and joint hypermobility. Similar to the paternal aunt, she can make a “praying sign behind her back.” She also has a history of recurrent fractures (including shoulder fractures requiring surgery). The proband’s mother does not have any history of EDS-like phenotypes, although she did report severe temporomandibular joint disorder. A maternal half-brother has scoliosis but no other bone or joint symptoms.

The mother (3380), father (1966) and affected male proband (2113) had their whole genome sequenced. For validation affected sister (0281) and half-brother (4736) of the proband were Sanger sequenced with the other three family members.

### DNA sample collection

Blood samples from the proband, sister, maternal half-brother and parents were collected by the Center for Applied Genomics at Children’s hospital of Philadelphia, with consent for prospective genomic analyses, access to EMRs, and re-contact for further studies.

### Whole genome sequencing and variant calling

**Fig. 2:**
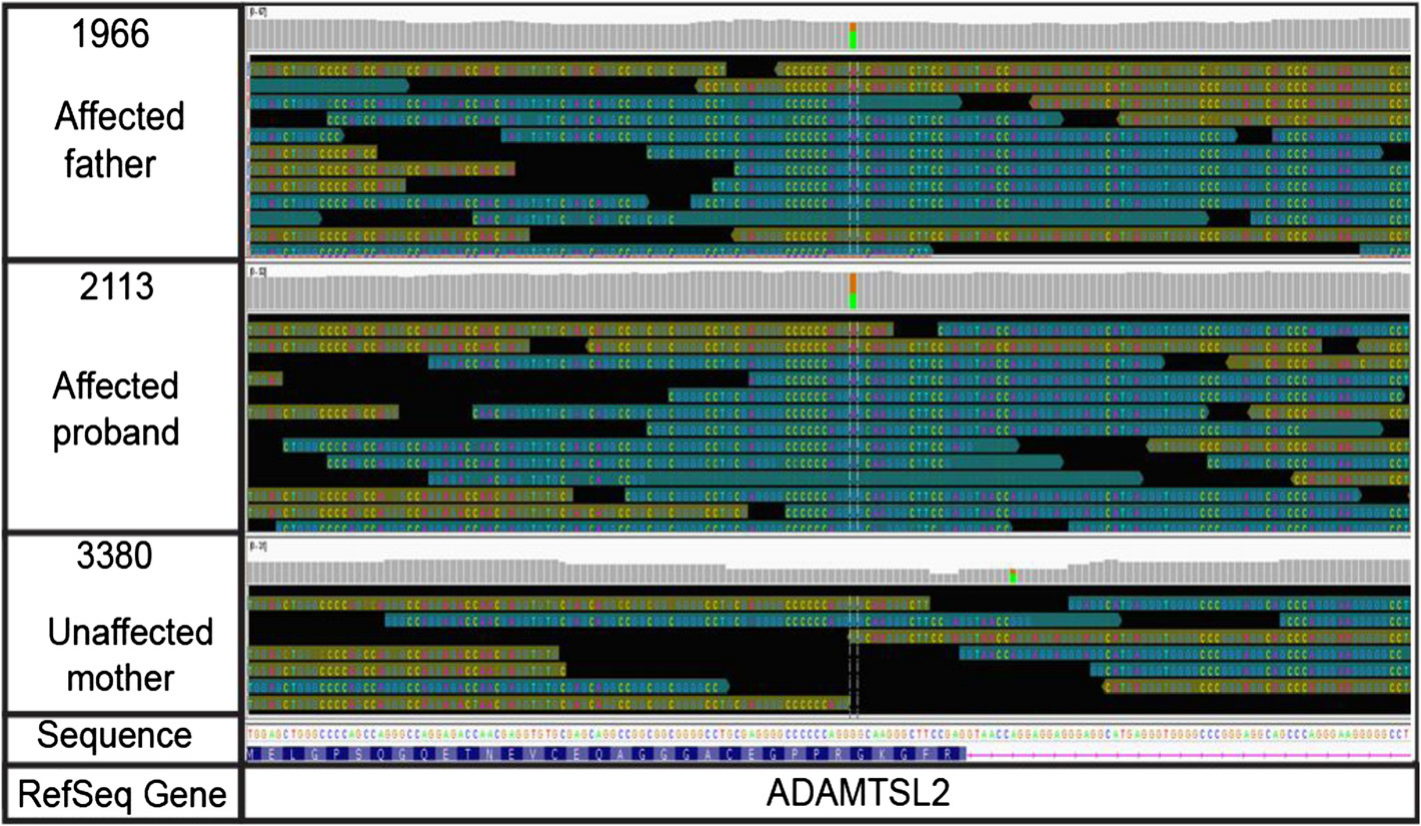
Reads of the Proband (2113), Father (1966) and Mother (3380) Summarized as a Coverage Plot. Position and significant number of mismatches with respect to reference are highlighted with colored bars. Individual base mismatch reads have been sorted and colored by strand

Genomic DNA was isolated from the blood samples by standard methods. Whole genome sequencing was performed using Illumina®HiSeq2000 on three of the members of family (proband and both parents) following the manufacturer’s instructions. All raw reads were aligned to the reference human genome build 37(UCSC hg19) using the Burrows–Wheeler alignment [9] (BWA, 0.6.2). Optical and PCR duplicates were marked and removed with Picard (v.1.73). Local realignment of reads in the indel sites and quality recalibration were performed with the Genome Analysis Tool Kit v1.6 [10]. SNPs and small INDELs were called with GATK UnifiedGenotyper [11]. ANNOVAR [12] and SnpEff [13] were used to annotate the variants. Fold coverage statistics are listed in (Table 1) and illustrated in (Fig. 2).

**Table 1:** WGS Coverage of Proband (2113), Father (1966) and Mother (3380)

SampleID	Average depth coverage	% of read depth 10×
2113	40	99
1966	40	98
3380	20	86

Average depth of coverage was 20–40×. Between 86 and 99 % reached a depth of 10×

WGS Coverage of Proband (2113), Father (1966) and Mother (3380). Average depth of coverage was 20–40x. Between 86 and 99 % reached a depth of 10x.

Initial variants were filtered following GATK best practice by excluding those variants where: 1) SNPs QualByDepth (QD) <2.0; 2) Root mean square (RMS) mapping quality (MQ) <40.0; 3) Phred-scaled p-value using Fisher’s exact test to detect strand bias (FS) > 60.0; 4) The haplotype score >13.0; 5) u-based z-approximation From Mann–Whitney Rank Sum Test for mapping qualities (MQRankSum) < −12.5; 6) ReadPosRankSum < −8.0.

Similarly, for indels, the following criteria were applied: 1) SNPs QualityByDepth (QD) <2.0; 2) ReadPosRankSum < −20.0; 3) InbreedingCoeff < −0.8; 4) Phred-scaled *p*-value using Fisher’s exact test to detect strand bias in reads (FS) >200.

### Variants validation through sanger sequencing

**Fig. 3:**
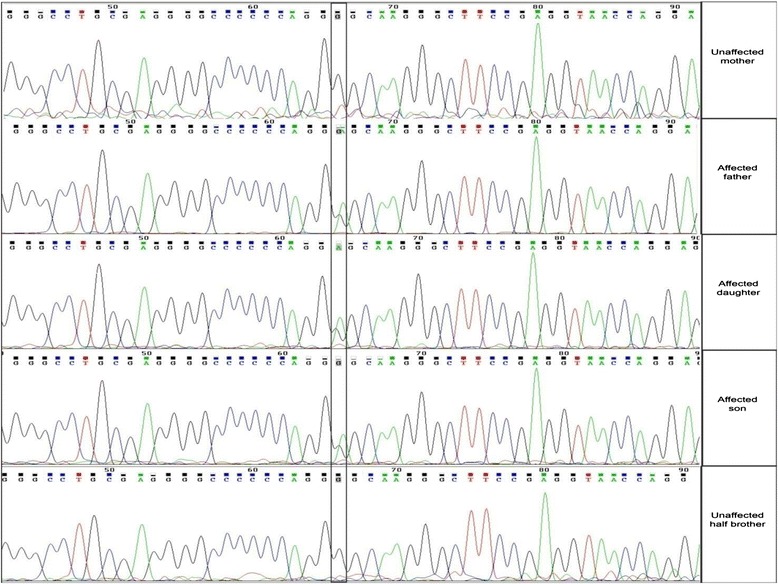
Sanger Validation of the proband and family members. Graphs show Sanger genotypes of the proband (G/A), unaffected mother (G/G), affected father (G/A), affected sister (G/A) and unaffected maternal half-brother (G/G)

Sanger sequencing of the variants (Fig. 3) was performed in order to confirm the heterozygous mutation identified by the variant detection analysis (see below). ABI 3730 sequencer with ABI BIgDye Terminator Cycle Sequencing kit was used. The reference genome sequence was obtained from UCSC Genome browser. The PCR primers were designed for the *ADAMTSL2* mutation using the PRIMER3 (http://bioinfo.ut.ee/primer3/) open source software. PCR was performed using the following primers: 1) *ADAMTSL2*-forward primer sequence: CAGGAGACCAACGAGGTGTG; 2) *ADAMTSL2*-reverse primer sequence: TGGCAGCTCTTAGGAACCTC. The resulting *. ABI files from the sequencer were loaded into ABI Sequence Scanner version 1.0 for further analysis. The sequence was manually reviewed to ensure reliability of genotype calls.

## Results

We recently identified an EDS patient, with Type VIIC form of EDS. Our screen for mutations in *ADAMTS2*, known to be associated with this sub-type and were negative. There were no mutations in any other known EDS-associated genes. Screenings for collagen/procollagen abnormalities were also negative. The patient’s family history includes a father and sister with EDS-like symptoms, as well as a number of other family members with EDS-type (endo) phenotypes. This pedigree information suggests an inheritance pattern consistent with autosomal dominant inheritance, possibly with variable expressivity.

Variant-calling produced 52 candidate loci, which are listed below. While *ADAMTSL2* is the most likely source of the EDS phenotype, one or more of these loci may be contributing to the disease pathogenesis.

**Fig. 4:**
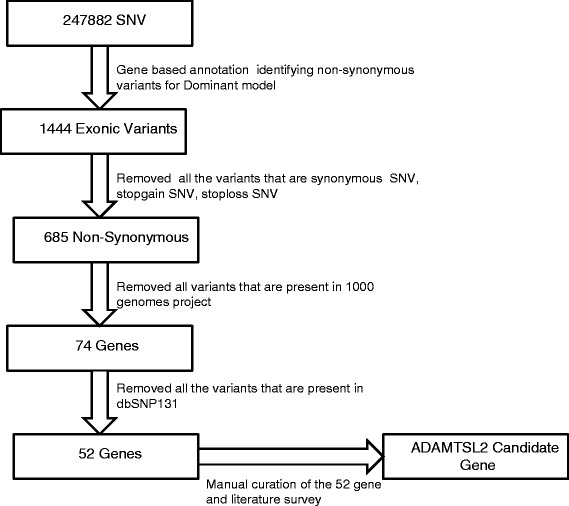
Pipeline Reduction Method for the Reduction of Variants. Manual curation identified mutations in *ADAMTSL2* as the most likely cause of the EDS phenotype

The whole genome analysis of proband (2113) yielded 3,546,583 variants. We identified about 248,772 SNV’s that are shared by the father, proband, and not present in the mother as we assumed it to be dominant model of inheritance. These were analyzed for non-synonymous mutations, which alter the amino acid sequence of a protein. We used ANNOVAR [12] for annotation of variants and used standard variant reduction method to reduce the number of candidate variants. As such we excluded variants that were in intronic regions, synonymous changes, and those identified by the dbSNP131 and the 1000 Genome Project [14]. This filtering resulted in a subset of novel non-synonymous candidate variants (Table 2). None of the known EDS genes (Table 3) showed mutations in the proband. However, manual curation and literature survey of the 52 variants resulted in the discovery of a mutation (c.1261G > A, p. Gly421Ser) in the *ADAMTSL2* gene (Table 4). As none of the other 52 variants resided in genes that were putatively candidates for causing EDS, we considered the (c.1261G > A, p. Gly421Ser) variant in the *ADAMTSL2* gene to be the most likely causative source of EDS in the family as it segregated in keeping with a disease-causing variant in this family. This heterozygous mutation (c.1261G > A, pGly421Ser) was detected in the proband (2113), affected father (1966) and, affected sister (0281). The mother (8380) and unaffected maternal half-brother (4376) were homozygous for the wild type allele. The mutation was confirmed by Sanger sequencing (Fig. 3). This process is illustrated in (Fig. 4).

**Table 2:** Variant-calling produced 52 candidate loci, which are listed below

Func	Gene	ExonicFunc	AAChange RefSeq	Conserved	Genomic location (hg19)	Ref	Het	Obs
exonic	NECAP2	non	NM_001145277: c.G527A: p.R176Q	336; LOD: 31	chr1:16778370	G	Het	A
exonic	GJB4	non	NM_153212: c.G371A: p.R124Q	387; LOD: 50	chr1:35227226	G	Het	A
exonic	ZC3H12A	non	NM_025079: c.C95T: p.P32L		chr1:37941192	C	Het	T
exonic	HEATR8	non	NM_001039464: c.T1103C: p.V368A		chr1:55119702	T	Het	C
exonic	PTGER3	non	NM_198718: c.A1136G: p.Q379R		chr1:71418711	T	Het	C
exonic	SLC22A15	non	NM_018420: c.G275C: p.S92T	483; LOD: 122	chr1:116534839	G	Het	C
exonic	FLG	stopgain	NM_002016: c.C9740A: p.S3247X		chr1:152277622	G	Het	T
exonic	IGSF9	non	NM_020789: c.G1085C: p.G362A	492; LOD: 133	chr1:159902414	C	Het	G
exonic	PLEKHA6	non	NM_014935: c.C29T: p.P10L		chr1:204242827	G	Het	A
exonic	OBSCN	non	NM_001098623: c.G6373T: p.A2125S		chr1:228464303	G	Het	T
exonic	RNF187	non	NM_001010858: c.C535T: p.R179W	439; LOD: 81	chr1:228680805	C	Het	T
exonic	EXO1	non	NM_003686: c.G820A: p.G274R	600; LOD: 366	chr1:242023882	G	Het	A
exonic	ITSN2	non	NM_019595: c.G4580A: p.R1527H		chr2:24431123	C	Het	T
exonic	FBXO41	non	NM_001080410: c.C1390T: p.R464C	609; LOD: 398	chr2:73492584	G	Het	A
exonic	BSN	non	NM_003458: c.C11090T: p.P3697L	510; LOD: 158	chr3:49700681	C	Het	T
exonic	HYAL3	non	NM_001200030: c.G533A: p.R178H	325; LOD: 28	chr3:50332501	C	Het	T
exonic	CRIPAK	non	NM_175918: c.C394G: p.H132D		chr4:1388693	C	Het	G
exonic	CRIPAK	non	NM_175918: c.C425G: p.P142R		chr4:1388724	C	Het	G
exonic	CRIPAK	syn	NM_175918: c.C456G: p.P152P		chr4:1388755	C	Het	G
exonic	TBC1D14	non	NM_001113361: c.C538G: p.L180V	569; LOD: 274	chr4:6925654	C	Het	G
exonic	SLC2A9	non	NM_020041: c.C824T: p.T275M		chr4:9922187	G	Het	A
exonic	RAB33B	non	NM_031296: c.C530T: p.T177M	653; LOD: 599	chr4:140394120	C	Het	T
exonic	FREM3	non	NM_001168235: c.G1570A: p.G524R	383; LOD: 48	chr4:144620259	C	Het	T
exonic	LOC345643	non	NM_001190787: c.A361T: p.T121S	419; LOD: 67	chr5:54518800	T	Het	A
exonic	GPR98	non	NM_032119: c.T6608C: p.V2203A	657; LOD: 625	chr5:89985795	T	Het	C
exonic	ULBP2	non	NM_025217: c.C79G: p.R27G		chr6:150263287	C	Het	G
exonic	SCAF8	non	NM_014892: c.T3450A: p.D1150E	678; LOD: 762	chr6:155154163	T	Het	A
exonic	IQCE	non	NM_152558: c.G528C: p.E176D	476; LOD: 115	chr7:2617938	G	Het	C
exonic	POU5F1B	non	NM_001159542: c.G142T: p.G48W		chr8:128428253	G	Het	T
exonic; splicing	ADAMTS13	non	NM_139026: c.A3817G: p.I1273V	378; LOD: 46	chr9:136324096	A	Het	G
exonic	ADAMTSL2	non	NM_001145320: c.G1261A: p.G421S	420; LOD: 68	chr9:136419800	G	Het	A
exonic	ARSD	non	NM_009589: c.C845A: p.A282D	329; LOD: 29	chrX:2835863	G	Het	T
exonic	SLC25A5	non	NM_001152: c.G413A: p.R138H	585; LOD: 319	chrX:118603925	G	Het	A
exonic	MAGEC1	non	NM_005462: c.G526C: p.V176L	358; LOD: 38	chrX:140993716	G	Het	C
exonic	SLC4A1	non	NM_000342: c.G539A: p.R180H		chr17:42337247	C	Het	T
exonic	HCN2	non	NM_001194: c.G227A: p.R76H	722; LOD: 1146	chr19:590172	G	Hom	A
exonic; splicing	UNC13A	non	NM_001080421: c.C3080T: p.P1027L	627; LOD: 471	chr19:17749893	G	Het	A
exonic	ZNF780B	non	NM_001005851: c.C1282T: p.R428C		chr19:40541484	G	Het	A
exonic	PSG8	non	NM_001130167: c.C110T: p.T37M		chr19:43268388	G	Het	A
exonic	ZC3H4	non	NM_015168: c.C3326T: p.P1109L	265; LOD: 16	chr19:47570199	G	Het	A
exonic	VSX1	stopgain	NM_199425: c.C165A: p.C55X		chr20:25062568	G	Het	T
exonic	WFDC11	non	NM_147197: c.T127C: p.W43R	295; LOD: 21	chr20:44278012	A	Het	G
exonic	LOC100132288	non	NM_001033515: c.C232T: p.R78C	422; LOD: 69	chr21:9909103	G	Het	A
exonic	SERPINB4	non	NM_002974: c.A193T: p.N65Y		chr18:61310424	T	Het	A
exonic	SEZ6L2	non	NM_001114099: c.G1342A: p.D448N	592; LOD: 341	chr16:29891206	C	Het	T
exonic	EPC1	non	NM_025209: c.G1384A: p.G462S	584; LOD: 314	chr10:32575629	C	Het	T
exonic	PWWP2B	non	NM_138499: c.G1036A: p.E346K	243; LOD: 13	chr10:134219040	G	Het	A
exonic	SGK223	non	NM_001080826: c.C622T: p.R208C	401; LOD: 57	chr8:8235297	G	Het	A
exonic	ATP6V0D2	non	NM_152565: c.G649A: p.D217N	513; LOD: 162	chr8:87162350	G	Het	A
exonic	ARPC1B	non	NM_005720: c.T1049G: p.M350R	410; LOD: 62	chr7:98991711	T	Het	G
exonic	CUL9	non	NM_015089: c.C2732T: p.P911L	463; LOD: 102	chr6:43164529	C	Het	T
exonic	XRN1	non	NM_001042604: c.A4484G: p.E1495G	622; LOD: 449	chr3:142037663	T	Het	C

While *ADAMTSL2* is the most likely source of the EDS phenotype, one or more of these loci may be contributing to the disease pathogenesis

**Table 3:** Adapted from Le Goff & Cormier-Daire (2008), Relevant Diseases Associated with THE ADAMTS(L) family

Gene	Function	Disease	Phenotype
*ADAMTS2*	Procollagen N propeptidase	EDS VIIC	Fragile skin, joint laxity
*ADAMTS10*	Unknown	Weill-Marchesani syndrome (WMS)	Short stature and extremities
			Thick skin
			Joint limitation
			Lens dislocation
*ADAMTS17*	Unknown	WMS-like syndrome 1	Short stature
			Lens dislocation
*ADAMTS13*	Von Willebrand factor cleaving-protease	Thrombotic thrombocytopenic purpura	Capilliaries and arterioles (heart, brain, kidney)
			Thrombosis of short stature and extremeties
			Joint limitation
			Cardiac vascular disease
*ADAMTSL4*	Associated with regulating deposition of *FBN1* into microfibrils	Ectopialentis	Lens dislocation
*ADAMTSL2*	Store/regulate latent TGFB in the extracellular matrix	EDS VIIC, Geleophysic dysplasia	Hypermobility, joint laxity

Mutations in *ADAMTS2* are associated EDS VIIC. A mutation in *ADAMTSL2* has previously been associated with geleophysic dysplasia, whose phenotype includes joint limitation

**Table 4:** Mutation characteristics SIFT polyphen scores and AA change

#Chrom	Position Hg19	Reference allele	Mutant allele	Gene	Type of mutation	Amino acid change	PolyPhen2 score	Sift score
Chr9	13641800	G	A	*ADAMTSL2*	Nonsynonymous	Gly421Ser	0.707	0.24

We identified a nonsynomous mutation resulting in an amino acid change—G1261A—at position 13641800 on chromosome 9

Mutation Characteristics SIFT Polyphen Scores and AA Change. We identified a nonsynomous mutation resulting in an amino acid change—G1261A—at position 13641800 on chromosome 9.

## Discussion

This study illustrates the success of the research strategy where by systematic data querying of phenotype data from a large biorepository can be cross-referenced with disease codes to identify the most likely cause of a rare disease. Because we are a research/treatment hub for children with rare disease such as EDS, our biorepository is disproportionately enriched for phenotypes such as these. As such, we anticipate this approach will resolve other rare and unidentified diseases in the future.

The A Disintegrin and Metalloproteinase with thrombospondin motifs (ADAMTS) family is associated with a range of biological processes, including formation/regulation of connective tissue structure, coagulation, arthritis, angiogenesis, and cell migration [15]. The ADAMTS superfamily includes 19 distinct members that are secreted enzymes and at least seven ADAMTS-like proteins without enzymatic activity [15–18]. The ADAMTS have two domains – the catalytic domain at the N terminus and the ancillary domain at their C terminus. These are required for interaction and are distinct from A Disintegrin and Metalloproteinases by 1+ type I repeats. They are synthesized as inactive zymogens, and post-translational modification is activated by their N-terminal propeptide, which is cleaved by furin [15]. ADAMTS-like proteins are different from ADAMTS ancillary domains in that they are lacking catalytic activity, due to the lack of protease domain.

Mutations in the ADAMTS family have been associated with EDS. Specifically *ADAMTS2* mutations are associated with Type VIIC, which is characterized by dermatosparaxis. This manifests in extreme skin fragility, spontaneous ruptures of internal organs, as well as premature rupture of membranes during pregnancy [19]. Colige et al. [20] were the first to identify mutations responsible for EDS VIIC, which were found in the gene encoding the procollagen protease *ADAMTS2*, located on chromosome 5qter (5q35.3) (originally identified under the alias procollagen I –Proteinase). They identified six EDS VIIC individuals, five of whom were homozygous for a C → T transition that resulted in a premature termination codon, Q225X, of which four were further homozygous at three downstream polymorphic sites (His 286 (CAT), Asp 676 (GAT), and Asp 844 (GAT)). A sixth patient was homozygous for premature termination codon, Q225X. These are all consistent with recessive inheritance.

Three additional mutations have been identified in the same gene in two patients: one had a homozygous deletion of exons 205, a second was compound heterozygous for a missing exon 3 (maternal) as well as a deletion that caused in-frame skipping of exons 14–16 [21]. This second patient constituted the first example of compound heterozygosity as the cause for EDS VIIC. Three more patients from the same study had variations found in normals, which was paralleled by silent functionality. All five EDS VIIC patients presented with facial dysmorphisms, hypermobility, skin and muscle fragility, and (at least) four of were born prematurely (~32–34 weeks). Recently, a newborn with EDS VIIC was described as presenting with congenital skull fractures and skin lacerations [19].

Adapted from Le Goff & Cormier-Daire (2008), Relevant Diseases Associated with THE ADAMTS (L) family. Mutations in *ADAMTS2* are associated EDS VIIC. A mutation in *ADAMTSL2* has previously been associated with geleophysic dysplasia, whose phenotype includes joint limitation

A mutation in *ADAMTSL2* has previously been associated with geleophysic dysplasia (GD) [OMIM: 231050], where the phenotype includes joint limitation. *ADAMTSL2* encodes a secreted glycoprotein that binds the cell surface and extracellular matrix, and interacts with latent transforming growth factor beta binding protein 1. GD is autosomally recessive, and can also include severe short stature, facial dysmorphism, a ‘happy’ face, progressive cardiac valvular thickening, respiratory insufficiency, and lysosomal-like storage vacuoles in different tissues.

Le Goff et al. [22] used in situ hybridization to human fetal tissue to demonstrate *ADAMTSL2* mRNA expression in cardiomyocytes, epidermis, dermal blood vessels, tracheal wall, skeletal muscle, pulmonary arteries, and bronchioles of the lung. Strong expression was also observed in chondrocyte columns in the hypertrophic and reserve zones of the proximal femoral growth plate. The group used a yeast 2-hybrid screen of a human muscle cDNA library, and found that *ADAMTSL2* bound to latent TGFβ-binding protein-1, which is important to regulating TGFβ availability and storing TGFβ in the extracellular matrix. While unsubstantiated by current medical records, there is a possible relationship here with the proband’s antibody deficiency and history of recurrent infection.

Allali S et al.[23] recently identified 33 patients, of whom 19 had a mutation in *ADAMTSL2*. The cohort collectively shared 13 mutations. The authors compared phenotypes in those that did/did not carry an *ADAMTSL2* mutation, finding that the main discriminating features were facial dysmorphism and tiptoe walking. These phenotypes were absent from the proband and his family. To our knowledge, *ADAMTSL2* is not associated with any other rare disorder.

We note that Table 1 also includes a number of loci that may also be associated with EDS and other phenotypes reported here. Of particular interest is a heterozygous non-synonymous single nucleotide variation in A disintegrin and metalloproteinase with a thrombospondin type 1 motif, member 13 (*ADAMTS13*), which is also known as von Willebrand factor-cleaving protease (VWFCP). Although this is an exonic splicing site, it may be of functional significance. *ADAMTS13* is a zinc-containing metalloprotease enzyme that cleaves von Willebrand factor (vWf)—a large protein involved in platelet adhesion and in aggregating vascular lesions.

While this demonstrates a success for data-querying of de-identified EMR data, we stress the importance of clinical phenotyping, which can only be accomplished on a local level. Nevertheless, we demonstrate the utility of unbiased large-scale recruitment and biobanking in deciphering the genetic etiology of rare Mendelian diseases. Families with most other Mendelian disorders for which genetic variants have been found were biasedly recruited by clinical experts. The majority of rare Mendelian diseases may go unsequenced under such a recruitment model. With unbiased large-scale clinical recruitment we strive to sequence as many rare Mendelian diseases as possible, and this work in uncovering a new mutation underlying EDS serves as a successful proof of concept to that effect.

## Conclusion

Although EDS type VII has been described as an autosomal-recessive disorder, the identification of heterozygous *ADAMTSL2* mutations demonstrates a dominant form of EDS, strictly fulfilling the diagnostic criteria for EDS VII. Future studies delineating the specific function of the *ADAMTSL2* protein will reveal how this novel mutation and others in N-glycan-rich module contribute to the disease phenotype.

### Consent

The proband and his family members consented to release of de-identified medical and genomic information for publication.

## Abbreviations

EDS: Ehler Danlos Syndrome
EMR: electronic medical record
OMIM: online mendelian inheritance in man

## References

[1] Beighton P, De Paepe A, Steinmann B, Tsipouras P, Wenstrup RJ (1998). Ehlers-Danlos syndromes: revised nosology, Villefranche, 1997. Ehlers-Danlos National Foundation (USA) and Ehlers-Danlos Support Group (UK). Am J Med Genet.

[2] Mayer K, Kennerknecht I, Steinmann B. Clinical utility gene card for: Ehlers-Danlos syndrome types I-VII and variants - update 2012. Eur J Hum Genet. 2013;21(1). doi:10.1038/ejhg.2012.162ejhg2012162 10.1038/ejhg.2012.162 PMC3533317 22892533

[3] De Paepe A, Malfait F (2012). The Ehlers-Danlos syndrome, a disorder with many faces. Clin Genet.

[4] Forlino A, Cabral WA, Barnes AM, Marini JC (2011). New perspectives on osteogenesis imperfecta. Nat Rev Endocrinol.

[5] Gajko-Galicka A (2002). Mutations in type I collagen genes resulting in osteogenesis imperfecta in humans. Acta Biochim Pol.

[6] Kocher MS, Shapiro F (1998). Osteogenesis imperfecta. J Am Acad Orthop Surg.

[7] Prockop DJ, Kuivaniemi H, Tromp G (1994). Molecular basis of osteogenesis imperfecta and related disorders of bone. Clin Plast Surg.

[8] Mundlos S, Spranger J (1991). Genetic disorders of connective tissues. Curr Opin Rheumatol.

[9] Li H, Durbin R (2010). Fast and accurate long-read alignment with Burrows-Wheeler transform. Bioinformatics.

[10] DePristo MA, Banks E, Poplin R, Garimella KV, Maguire JR, Hartl C (2011). A framework for variation discovery and genotyping using next-generation DNA sequencing data. Nat Genet.

[11] McKenna A, Hanna M, Banks E, Sivachenko A, Cibulskis K, Kernytsky A (2010). The Genome Analysis Toolkit: a MapReduce framework for analyzing next-generation DNA sequencing data. Genome Res.

[12] Wang K, Li M, Hakonarson H (2010). ANNOVAR: functional annotation of genetic variants from high-throughput sequencing data. Nucleic Acids Res.

[13] De Baets G, Van Durme J, Reumers J, Maurer-Stroh S, Vanhee P, Dopazo J (2012). SNPeffect 4.0: on-line prediction of molecular and structural effects of protein-coding variants. Nucleic Acids Res.

[14] Abecasis GR, Altshuler D, Auton A, Brooks LD, Durbin RM, Gibbs RA (2010). A map of human genome variation from population-scale sequencing. Nature.

[15] Le Goff C, Cormier-Daire V (2011). The ADAMTS(L) family and human genetic disorders. Hum Mol Genet.

[16] Kuno K, Kanada N, Nakashima E, Fujiki F, Ichimura F, Matsushima K (1997). Molecular cloning of a gene encoding a new type of metalloproteinase-disintegrin family protein with thrombospondin motifs as an inflammation associated gene. J Biol Chem.

[17] Apte SS (2009). A disintegrin-like and metalloprotease (reprolysin-type) with thrombospondin type 1 motif (ADAMTS) superfamily: functions and mechanisms. J Biol Chem.

[18] Apte SS (2004). A disintegrin-like and metalloprotease (reprolysin type) with thrombospondin type 1 motifs: the ADAMTS family. Int J Biochem Cell Biol.

[19] Solomons J, Coucke P, Symoens S, Cohen MC, Pope FM, Wagner BE (2013). Dermatosparaxis (Ehlers-Danlos type VIIC): prenatal diagnosis following a previous pregnancy with unexpected skull fractures at delivery. Am J Med Genet A.

[20] Colige A, Sieron AL, Li SW, Schwarze U, Petty E, Wertelecki W (1999). Human Ehlers-Danlos syndrome type VII C and bovine dermatosparaxis are caused by mutations in the procollagen I N-proteinase gene. Am J Hum Genet.

[21] Colige A, Nuytinck L, Hausser I, van Essen AJ, Thiry M, Herens C (2004). Novel types of mutation responsible for the dermatosparactic type of Ehlers-Danlos syndrome (Type VIIC) and common polymorphisms in the ADAMTS2 gene. J Invest Dermatol.

[22] Le Goff C, Morice-Picard F, Dagoneau N, Wang LW, Perrot C, Crow YJ (2008). ADAMTSL2 mutations in geleophysic dysplasia demonstrate a role for ADAMTS-like proteins in TGF-beta bioavailability regulation. Nat Genet.

[23] Allali S, Le Goff C, Pressac-Diebold I, Pfennig G, Mahaut C, Dagoneau N (2011). Molecular screening of ADAMTSL2 gene in 33 patients reveals the genetic heterogeneity of geleophysic dysplasia. J Med Genet.

